# Wnt pathway is involved in 5-FU drug resistance of colorectal cancer cells

**DOI:** 10.1038/s12276-018-0128-8

**Published:** 2018-08-14

**Authors:** Lingfeng He, Hong Zhu, Shiying Zhou, Ting Wu, Huan Wu, Huan Yang, Huiwen Mao, Chandra SekharKathera, Avilala Janardhan, Ashlin M. Edick, Anna Zhang, Zhigang Hu, Feiyan Pan, Zhigang Guo

**Affiliations:** 1 Jiangsu Key Laboratory for Molecular and Medical Biotechnology, College of Life Sciences, Nanjing Normal University, 1 WenYuan Road, Nanjing, 210023 China; 2 Department of Animal Biosciences, University of Guelph, Guelph, ON Canada; 3 Department of Psychology, University of Guelph, Guelph, ON Canada

## Abstract

Colorectal cancer (CRC) is one of the leading causes of cancer-related death worldwide. 5-Fluorouracil (5-FU) is widely used in the treatment of cancers, but its antineoplastic activity is limited in drug-resistant cancer cells. To investigate the detailed mechanism of 5-FU resistance, we developed a model of 5-FU-resistant cells from HCT-8 cells, a well-established colorectal cancer cell line. We found that the drug-resistant cells demonstrated high expression of TCF4 and β-catenin, indicating an upregulated Wnt pathway. A microarray analysis revealed that the suppression of the checkpoint kinase 1 (CHK1) pathway explained the resistance to 5-FU, especially in p53 wild-type cancer cells such as HCT-8. Our data also demonstrated that the CHK1 pathway is suppressed by the Wnt pathway in 5-FU-resistant cells. In summary, we have discovered a novel mechanism for 5-FU resistance mediated by histone deacetylation, which also revealed the crosstalk between the Wnt pathway and CHK1 pathway.

## Colorectal cancer: pathways to drug resistance

The interaction between two signaling pathways may be related to resistance to a leading cancer drug in colorectal cancer cells. The drug 5-fluorouracil (5-FU) is widely used to limit malignant cell proliferation, but some cancers, including colorectal cancers, are resistant to 5-FU. Zhigang Guo at Nanjing Normal University, China, and co-workers have uncovered the signaling pathways behind 5-FU resistance in colorectal cancer cell lines. They found that 5-FU-resistant cells expressed high levels of two proteins indicative of an over-active Wnt signaling pathway, a crucial pathway in healthy development, the disruption of which is implicated in many diseases. This upregulated pathway suppressed another signaling pathway, leading to reduced cancer cell death. This interaction between the pathways could prove to be a valuable therapeutic target to limit resistance to 5-FU.

## Introduction

Although considerable progress has been made in the treatment of colorectal cancer (CRC) in recent years, it remains as one of the leading causes of cancer-related death worldwide^1^. To date, 5-Fluorouracil (5-FU) remains a commonly used chemotherapeutic drug in cancer treatments and clinical studies^2^. Over the past decades, an increased understanding of the 5-FU mechanism has promoted the progress of new strategies that increase antineoplastic activity. The antineoplastic efficacy of 5-FU is attributed to its ability to increase DNA damage, which results in cell growth arrest and apoptosis. However, clinical efficacy is reduced due to the chemotherapeutic drug resistance of cancer cells. Despite extensive research in recent years, drug resistance remains a critical limitation to the clinical application of 5-FU and related chemotherapeutic drugs^3^. Thus, further exploration on overcoming the chemotherapeutic drug resistance of cancer cells would be instrumental in increasing the potency of cancer therapy^4^.

The DNA damage response is initiated by molecular complexes or pathways, including ATM and ATR^5^. The DNA damage response activates the checkpoint network, which regulates the cell cycle transition, DNA repair, and cell apoptotic response. As previously known, tumor suppressor p53 maintains DNA integrity by transcriptionally activating downstream target genes such as *P21* and *GADD45b*, which induces cell cycle arrest in response to DNA damage^6^. Previous reports suggested that 5-FU can activate the p53 signal through several mechanisms, including inhibition of thymidylate synthase (TS) by FdUMP, which results in DNA damage^7^.

CHK1 plays a critical role in the checkpoint activation pathway^8^. In response to DNA damage, CHK1 activates p53, which induces the phosphorylation and stabilization by ATR at the serine residue^9,10^. Upon activation, CHK1 phosphorylates a series of downstream targets^11^, such as CDC25a and CDC25c, resulting in activation of DNA damage checkpoints, cell cycle arrest, DNA repair, and/or p53-induced apoptosis^12^. Loss-of-function CHK1 mutations have been reported in stomach, endometrial, and CRCs^13,14^.

DNA-damaging reagents such as 5-FU are the most commonly used chemotherapy drugs for clinical cancer therapy, as they induce cell cycle arrest to prevent cell proliferation and trigger cell apoptosis in cancer cells^15^. The therapeutic effect of chemotherapy drugs is highly dependent on the status of TP53 in cancer cells, which is concerning as p53 pathway mutations occur frequently in human cancer^16,17^. It has been reported that over 60% of cancer cells harbor somatic mutations in TP53^18^. The mechanism by which p53-normal cancer cells generate resistance to apoptosis induced by DNA damage reagents and chemotherapy drugs is not well understood.

To study the detailed mechanism, we established drug-resistant cells from a CRC cell line and performed a microarray analysis. We found that Wnt signal activation confers 5-FU resistance in HCT-8R cells by suppressing the CHK1 pathway in TP53 wild-type cells such as HCT-8. Our data revealed that histone modification plays a critical role in the regulation of the CHK1 pathway^19,20^. Our paper contributes to the understanding of the crosstalk between the Wnt pathway and the p53-regulated apoptotic pathway, which will bring us a step closer to the mechanism of drug resistance in cancer cells.

## Materials and methods

### Cell culture

All cell lines used in this study were obtained from American Type Culture Collection (Maryland, USA) and cultured under conditions as directed by the product instructions. The 5-FU-resistant HCT-8 cells (HCT-8R) were selected and established from HCT-8 cells treated with stepwise increased concentrations of 5-FU (0, 0.01, 0.1, 0.5, 2, and 10 µM) over 5 months. The acquired drug-resistant cells were cultivated and stabilized in 10 µM 5-FU-containing medium. HCT-8 cells were cultured in RMPI 1640 supplemented with 10% fetal bovine serum (Invitrogen) and penicillin-streptomycin (Invitrogen) at 37 °C in a humidified 5% CO_2_ incubator.

### RNA extraction

Total RNA was extracted from cultivated cells using TRIzol reagent (Invitrogen, Carlsbad, CA, USA) in accordance with the manufacturer’s protocols. RNA purification was performed according to the manufacturer’s instructions for the RNeasy Mini Kit (Qiagen).

### Quantitative PCR analysis

Total mRNA was isolated using TRIzol reagent (Life Technologies, Carlsbad, CA, USA). Reverse transcription reaction was performed according to the manufacturer’s instructions. Quantitative PCR (Q-PCR) was performed using a reaction mixture with SYBR Q-PCR Super Mix-UDG (Invitrogen, Carlsbad, CA, USA). All PCR amplifications were performed in triplicate and repeated in three independent experiments. The primer sequences are shown in supplemental Table 1.

### Gene chip

Microarray experiments were performed using 10 mg total RNA; details of reverse transcription, labeling, and hybridization were according to the manufacturer’s protocol. Briefly, the procedures included first-strand cDNA synthesis, second-strand cDNA synthesis, double-stranded cDNA clean up, in vitro transcription, cDNA purification, and fragmentation. A total of 20 mg biotinylated cRNA was used for each array hybridization. Samples were hybridized onto Affymetrix 3′IVT Microarrays (Affymetrix, Santa Clara, CA, USA). After hybridization at 45 °C overnight, arrays were subsequently developed with phycoerythrin-conjugated streptavidin using a fluidics station (GeneChip Fluidics Station 450, Santa Clara, CA, USA) and were scanned to obtain quantitative expression levels. Paired tumor and normal tissue specimens from each patient were processed simultaneously throughout the experimental process.

### Microarray analysis

Pathway-level gene expression changes were identified using gene-set enrichment analysis. Annotated gene sets for Kyoto Encyclopedia of Genes and Genomes (KEGG) pathways were downloaded from the Molecular Signatures Database. Expression changes for genes and KEGG pathways were evaluated using linear models for microarrays and gene-set variability analysis. A *P*-value cutoff of 0.05 was applied after false discovery rate correction. MAS3.0, significance analysis of microarray *q*-value ≤ 5% and fold change ≥ 2 or ≤0.5 were applied; Affymetrix^®^GeneChip^®^ Command Console^®^ Software.

### Immunofluorescence

Cells were cultured in six-well plates containing acid-treated cover slides and incubated overnight. The cover slides were washed with phosphate-buffered saline (PBS), fixed with 4% formaldehyde in PBS for 30 min, and then washed with PBS. Triton X-100 (0.2%) was added for 10 min to permeabilize the cells. Slides were blocked with 3% bovine serum albumin and then incubated with primary antibody. The slides were washed and then incubated with secondary antibody conjugated with fluorescein isothiocyanate, followed by PBST washing and 4′,6-diamidino-2-phenylindole (DAPI) staining. The mounted slides were viewed with a Zeiss Axioscope, and images were captured with a charge-coupled-device camera.

### Cell viability assay

Cells were seeded in 12-well plates and then incubated. After different treatments, the number of viable cells was determined by the Countstar Automated Cell Counter. At least three replicas for each clone were averaged. Data were expressed as percentage of growth relative to untreated cells.

### Flow cytometric analysis of apoptosis

A total of 1 × 10^6^ cells was trypsinized, washed, and resuspended in 1 mL PBS with 5% fetal bovine serum. Subsequently, cells were washed twice with ice-cold PBS, and then 3 mL ice-cold ethanol was added to fix the samples. After centrifugation, cells were resuspended in 1 mL of 50 μg/mL RNase A and 50 μg/mL propidium iodide at 37 °C for 30 min. Then, the apoptosis ratio was analyzed using a fluorescence-activated cell sorting (FACS) flow cytometer (FACS Calibur, BD).

### TUNEL assay

Cells were cultured in six-well plates containing acid-treated cover slides and incubated overnight. The cover slides were then washed with PBS, fixed with 4% formaldehyde in PBS for 30 min, and washed with PBS again. Triton X-100 (1%) was added for 5 min to permeabilize the cells. Three percent H_2_O_2_ was then added for 10 min, and cover slides were washed twice with ice-cold PBS. Cells were incubated with TdT marker solution at 37 °C for 1 h and then gently washed with PBS three times. Cells were incubated in the dark with 100 μL dyeing buffer solution for 30 min, washed with PBS, and stained with DAPI.

### Western blotting analysis

Cells were harvested from the plates and resuspended in lysis buffer. Protein concentrations were determined with the Bio-Rad protein assay system. Aliquots of cell extracts containing 30 µg proteins were separated by SDS-polyacrylamide gel electrophoresis on 12% gels and transferred to polyvinylidene difluoride membranes. The membranes were blocked for 1 h with blocking buffer (5% nonfat milk powder in PBS). The membranes were incubated with primary and secondary antibodies, followed by chemiluminescence detection using ECL Western blotting detection reagents (Pierce Bio.).

### Antibodies

Anti-p53 antibody (SC-126), anti-Caspase-3 (SC-7148), anti-APE1 (SC-55498), and anti-β-catenin (SC-7963) were purchased from Santa Cruz. Anti-CHK1 antibody (#2360) was obtained from CST Biotechnology. Anti-Vinculin antibody (MAB3574) and active-β-catenin (MAB-05665) were obtained from Millipore. Anti-Polβ (ab26343) was obtained from Abcam. Anti-FEN1 (70185) was purchased from Gentex. Anti-TBB5(AM103a), anti-GAPDH (0063), and anti-β-Actin (M0001) were obtained from Abgent.

### Small interfering RNA oligonucleotides

The CHK1 small interfering (siRNA) oligonucleotides were purchased from Santa Cruz. Cells were transfected with the indicated oligonucleotide using the Oligofectamine reagent (Invitrogen). After 48 h of siRNA transfection, cells were used for functional assays, and the remaining cells were harvested for western blotting analysis.

### Statistical analysis

The statistical significance of the differences between various groups in the same experiments was determined by standard deviation (SD) and Student’s *t*-test for multiple comparisons. Statistical significance was assumed when *P* < 0.05. All experiments were repeated at least twice to confirm reproducibility. All data are displayed as the mean ± SD.

## Results

### Establishment of 5-FU-resistant CRC cells

**Fig. 1:**
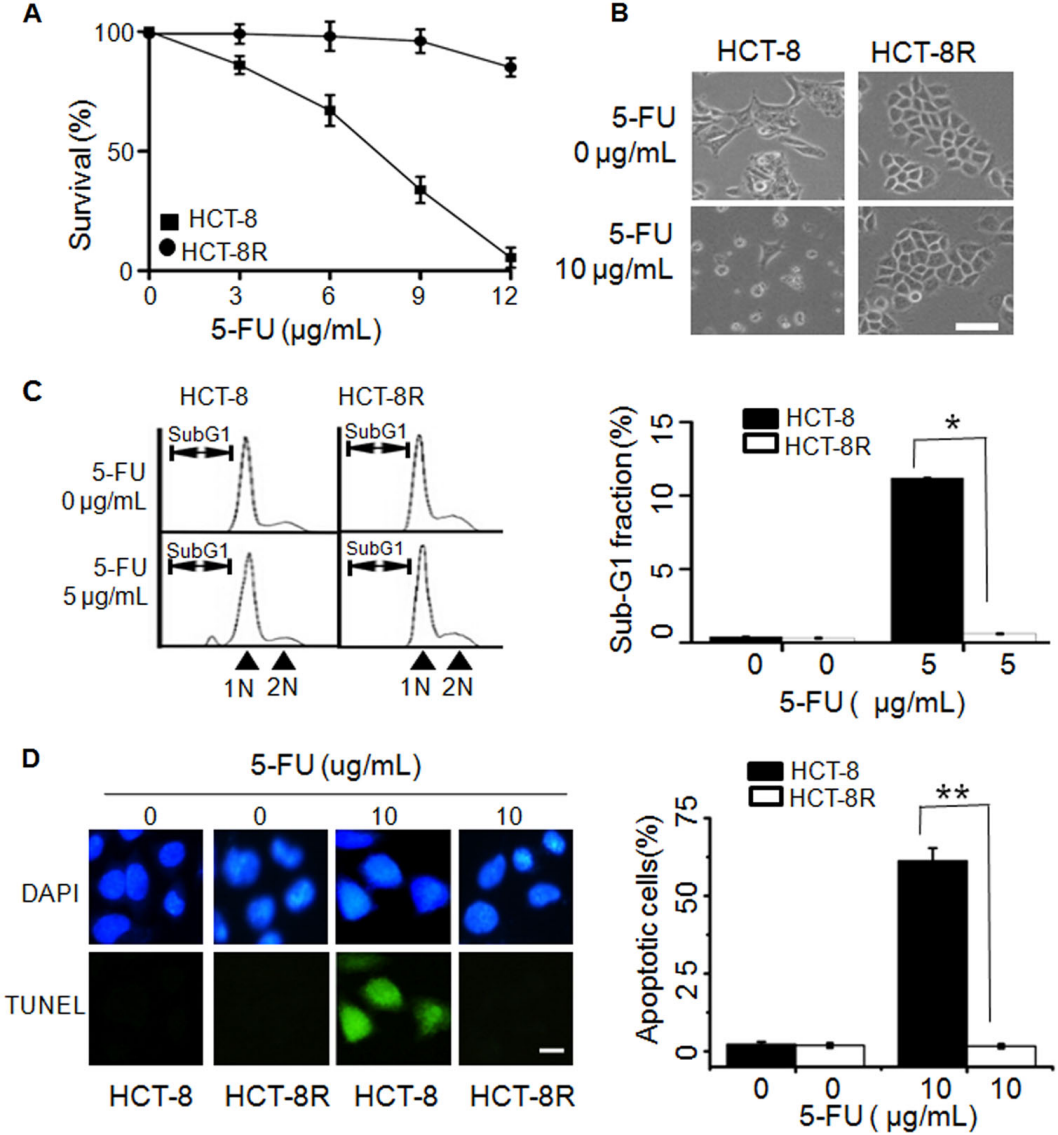
Generation and characterization of 5-FU-resistant cells from HCT-8 cells. **a** Growth curve of HCT-8R and HCT-8 cells under 5-FU treatment. The data represent the means ± SD from three independent experiments. **b** Cell morphology of HCT-8R and HCT-8 cells (×400). Scale bar, 100 µm. **c** The result of FACS analysis shows the subG1 peak of HCT-8R and HCT-8 cells under treatment with 5 µg/mL 5-FU for 24 h. The FACS result shows the apoptotic ratio of cells induced by 5-FU treatment. HCT-8 WT control cell and HCT-8R resistant cell ratios were 8.1% and 0.2%, respectively. The data represent the means ± SD from three independent experiments. *P < 0.05. **d** TUNEL assay shows the apoptotic HCT-8 cells under 10 µg/mL 5-FU treatment for 48 h. Scale bar, 10 µm. The data represent the means ± SD from three independent experiments. **P < 0.01

To obtain 5-FU-resistant cells, parental HCT-8 colorectal cells were treated with increasing concentrations of 5-FU. Clone cells with 5-FU resistance, labeled HCT-8R, showed acquired resistance to 5-FU by cell survival assay (Fig. 1a). At the concentration of 10 µg/mL 5-FU, while most of the parental HCT-8 cells were killed by 5-FU, HCT-8R displayed normal morphology (Fig. 1b). FACS and TUNEL assay results showed a decreased proportion of subG1 cells (Fig. 1c) or TUNEL-stained cells (Fig. 1d) compared to parental HCT-8 cells. This indicates that HCT-8R cells are resistant to 5-FU-induced cell apoptosis.

### 5-FU resistance in HCT-8R cells is not related to the base excision repair pathway

5-FU has been reported to cause DNA base lesions in the base excision repair (BER) pathway^21^. Therefore, when drug resistance is elevated in HCT-8R, an elevation in BER activity should also be observed. However, when we examined the protein level of essential enzymes involved in the BER pathway, the results showed insignificant changes in enzymatic expression levels in APE1, Polβ, and FEN1 (Figure S1A)^22,23^. When BER was reconstituted with cell extracts from HCT-8 and HCT-8R cells, there was no significant difference in terms of DNA repair efficiency between HCT-8 and HCT-8R cells (Figure S1B). These results indicate that the BER pathway is not involved in the 5-FU resistance of HCT-8R cells.

### Identification of genes associated with 5-FU response in the HCT-8R cells

**Fig. 2:**
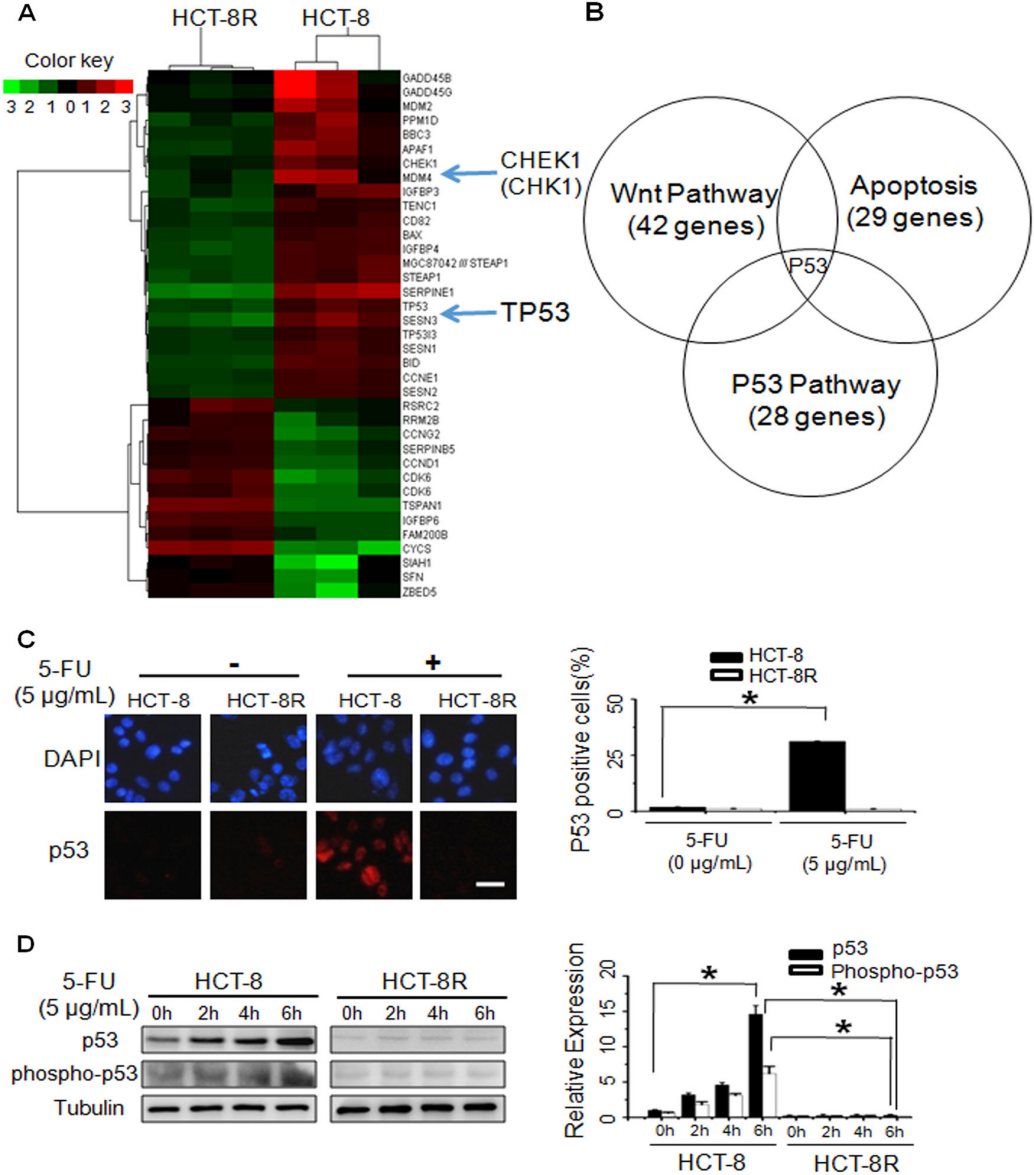
Gene expression signature associated with 5-FU resistance. **a** Part of the hierarchical cluster diagram of HCT-8R cells vs. HCT-8 cells is shown. **b** Venn diagram of genes that were differentially expressed in HCT-8R cells. The gene expression file and KEGG analysis results of the p53-related apoptosis pathway, cell cycle regulation pathway, and Wnt pathway were analyzed. **c** The 5-FU-induced expression of p53 was examined by immunostaining. Shown are the representative images of 5-FU-treated HCT-8 or HCT-8R cells. Scale bar, 50 µm. The data represent the means ± SD from three independent experiments. *P < 0.05. **d** The induction of p53 expression in 5-FU-treated HCT-8 cells was confirmed by immunoblot. The 10 µg/mL 5-FU treatment induced p53 expression in HCT-8 WT cells but not in HCT-8R cells. *P < 0.05

To identify potential genes involved with the response to 5-FU in HCT-8R cells, RNA was extracted from each cell line. Gene expression was analyzed using an Affymetrix 3′IVT Microarray. Gene expression was compared by pairwise subtraction of normalized expression levels for each gene to determine differential expression between 5-FU-resistant HCT-8R and parental control HCT-8 cells (Fig. 2a and Supplementary Figure S2). Pathway analysis revealed that the p53 pathway, the apoptosis pathway, and the Wnt pathway were affected in HCT-8R cells. Among the affected genes, p53 was the common protein involved in all three pathways (Fig. 2b). The mRNA level and the protein level of TP53 were significantly decreased in HCT-8R cells (Figure S3A and S3B).

### p53 is downregulated and inactivated in HCT-8R cells

p53 plays a major role in cellular responses to genomic aberrations, such as DNA damage^24^. Activation and stabilization of p53 lead to cell cycle arrest, DNA repair, or apoptosis^25^. Generally, 5-FU-induced DNA damage increases p53 phosphorylation^26^. To evaluate the HCT-8 and HCT-8R cell response to DNA damage, the cells were treated with 5 µg/mL 5-FU. Then, the p53 protein level was determined by immunofluorescence (Fig. 2c) and western blotting assay (Fig. 2d). The results showed that the total protein and phosphorylation levels of p53 were affected by 5-FU in HCT-8 cells but not in HCT-8R cells. These data suggest that the resistance of HCT-8R to 5-FU is due to the inactivation of p53-related apoptosis pathways.

CHK1 acts as an upstream regulator of p53 in the checkpoint pathway. Therefore, we speculated that the inactivation of the p53 pathway observed in HCT-8R cells resulted from CHK1 knockdown or inactivation. The results showed that both CHK1 protein and its phosphorylated form were reduced in HCT-8R cells compared to HCT-8 cells (Figure S3B). Downregulation of CHK1 reduced total and phosphorylated p53 protein levels (Figure S3C), while overexpression of CHK1 increased both the total and phosphorylated protein forms of p53 (Figure S3D). These data suggest that manipulation of CHK1 in cells could alter the cellular response to 5-FU.

### CHK1 is a determinant of 5-FU resistance in HCT-8R cells

**Fig. 3:**
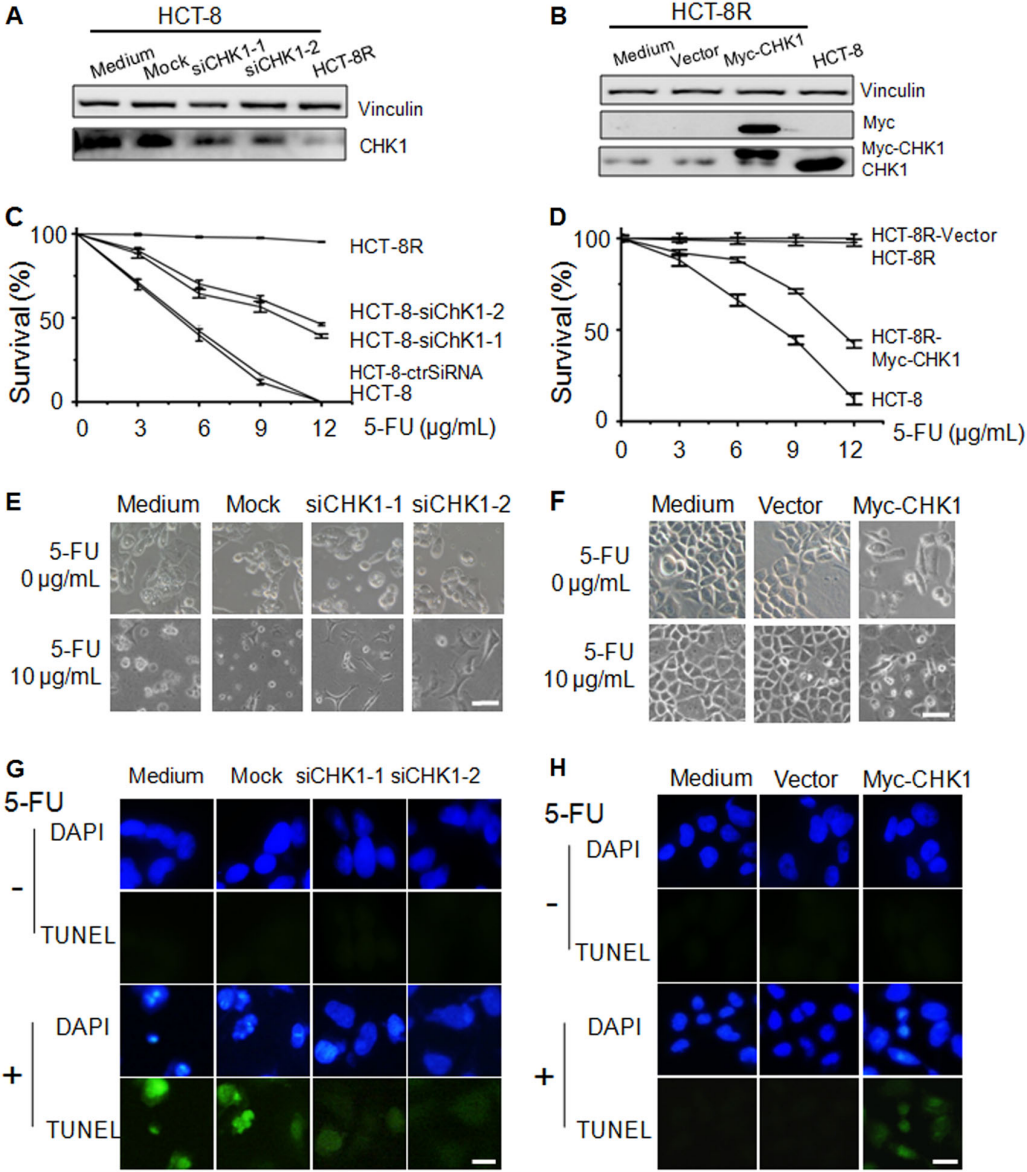
Enhanced resistance to 5-FU is due to the suppressed checkpoint pathway. **a** The knockdown efficiency of CHK1 was shown by immunoblot assay. **b** Overexpressed CHK1 expression was confirmed by immunoblotting assay. **c** The cell survival curve shows that the cell survival rate of the CHK1 knockdown HCT-8 cells with 10 µg/ml 5-FU was significantly higher than that of mock-transfected cells. **d** Overexpressed CHK1 expression decreased the cell survival rate in 10 µg/mL 5-FU-containing medium. **e** Cell morphology under a microscope. All cells transfected with CHK1 siRNA were treated with 10 µg/mL 5-FU for 48 h. Scale bar, 50 µm. **f** Cell morphology of CHK1-overexpressing HCT-8 cells and mock-treated cells (×400). Scale bar, 50 µm. **g** TUNEL assay shows the apoptotic cells induced by 5-FU treatment. Scale bar, 10 µm. **h** TUNEL assay shows the apoptotic cells induced by CHK1 overexpression. Scale bar, 10 µm

Because CHK1 is downregulated in 5-FU-resistant cells, we speculated that manipulation of CHK1 could alter the response of cancer cells to 5-FU. To confirm this, the CHK1 expression level was manipulated in cells by siRNA (Fig. 3a) and CHK1 overexpression (Fig. 3b). The survival rate of cells was determined after exposure to 5-FU. We found that CHK1-knockdown cells showed higher resistance to 5-FU (Fig. 3c), whereas CHK1-overexpressing cells demonstrated greater sensitivity than control cells to this drug (Fig. 3d). The above data proved that manipulation of the CHK1 level partially altered the sensitivity of cancer cells to 5-FU. To confirm the above data, the cell morphologies of CHK1-knockdown cells (Fig. 3e) and CHK1-overexpressing cells were recorded (Fig. 3f). To confirm whether CHK1 levels could impact cell apoptosis, we performed TUNEL assays after treatment with 5-FU. CHK1 knockdown prevented cell apoptosis induced by 5-FU (Fig. 3g), whereas overexpression of CHK1 augmented apoptosis induced by 5-FU (Fig. 3h). These data indicate that CHK1 regulates the sensitivity of cancer cells to 5-FU treatment.

### Wnt pathway is upregulated in HCT-8R cells

**Fig. 4:**
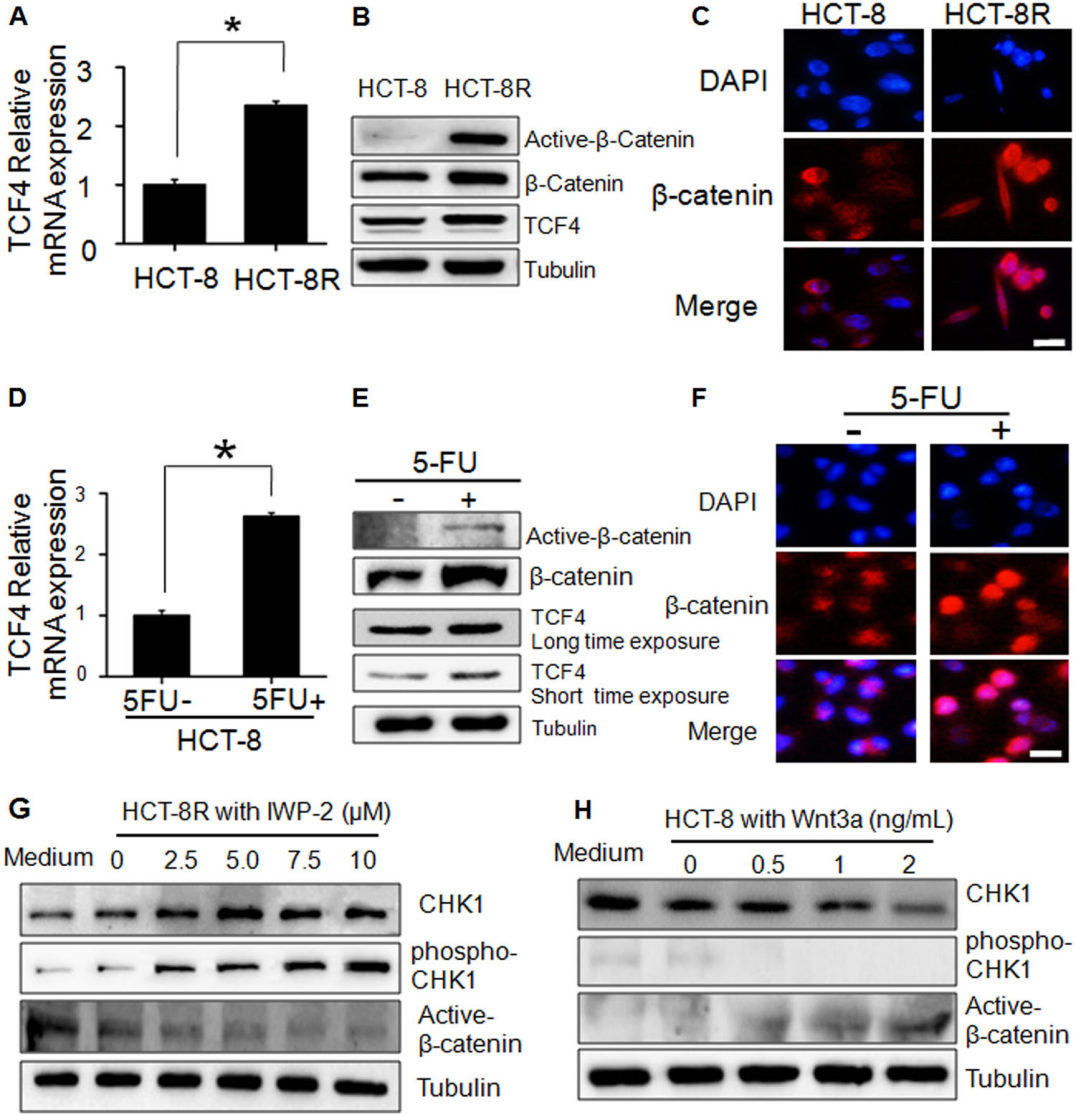
5-FU-resistant cells are Wnt pathway-activated cells. **a** TCF4 mRNA expression was elevated in HCT-8R cells. The data represent the means ± SD from three independent experiments. *P < 0.05. **b** The elevated expression of TCF4, β-catenin, and active-β-catenin in HCT-8R cells was examined by immunoblot. **c** Immunofluorescence detection of β-catenin HCT-8R cells. Scale bar, 20 μm. **d** TCF4 mRNA expression was elevated in 5-FU-treated HCT-8 cells. *P < 0.05. **e** The expression of TCF4, β-catenin, and active-β-catenin in HCT-8 cells (induced by 100 µg/mL 5-FU for 48 h) was examined by western blotting assay. **f** Immunofluorescence detection of β-catenin HCT-8 cells. Scale bar, 20 μm. **g** IWP-2 efficiently suppresses the expression of active-β-catenin, while CHK1 and p-CHK1 were upregulated in HCT-8R cells. **h** Wnt3a stimulated the expression of active-β-catenin, while CHK1 and p-CHK1 expression were suppressed by the enhanced Wnt signal

The mechanism involved in suppressing the CHK1 pathway in 5-FU-resistant cells (HCT-8R) is still unclear. As shown in Fig. 2b, the Wnt pathway was affected in HCT-8R cells. In particular, the RNA microarray data showed that the expression of TCF4, a key element in the Wnt pathway, was significantly upregulated (Fig. 4a). Both total β-catenin and active-β-catenin expression were upregulated in HCT-8R cells, further confirming the activation of the Wnt pathway in HCT-8R cells (Fig. 4b). Active-β-catenin activates the Wnt pathway by detecting non-phosphorylated β-catenin, which enters the nuclei to transcriptionally activate the downstream target genes of the Wnt/β-catenin pathway. The translocation of activated β-catenin was also shown by immunofluorescence staining in HCT-8R cells, as indicated in Fig. 4c. The above data indicate that HCT-8R cells are Wnt pathway-activated cells. The same procedure was repeated in HCT-8 cells. The analyzed results demonstrated that increases in both the TCF4 mRNA and protein levels were induced under 5-FU treatment (Fig. 4d, e). Both total β-catenin and active-β-catenin expression were upregulated in HCT-8 cells upon 5-FU stimulation. The nuclear aggregation of β-catenin was confirmed by immunofluorescence staining in HCT-8 cells exposed to 5-FU (Fig. 4f).

### Wnt pathway suppresses the CHK1-p53 pathway in HCT-8 cells

Next, we wondered whether crosstalk existed between the CHK1 and Wnt pathways in HCT-8R cells. We found that neither downregulation nor overexpression of CHK1 in cells affected the activity of the Wnt pathway (data not shown). Therefore, we looked into whether the Wnt pathway could regulate CHK1 and showed that the cellular localizations of β-catenin and CHK1 protein were exclusive (Figure S4). Next, we treated HCT-8R cells with IWP-2, a well-established inhibitor of the Wnt pathway. As shown in Fig. 4g, the CHK1 pathway was suppressed by the Wnt pathway in HCT-8R cells. Next, we performed a Wnt signal stimulation experiment in HCT-8 WT cells (Fig. 4h). We found that the Wnt signal suppressed CHK1 protein expression and phospho-CHK1 protein levels in a dose-dependent manner.

### Modulating Wnt signaling activity controls drug resistance in HCT-8R cells

To reinforce the idea that the alteration of Wnt activity would lead to a change in drug resistance, we designed further experiments to study the effect of Wnt signaling on HCT-8 cells.

**Fig. 5:**
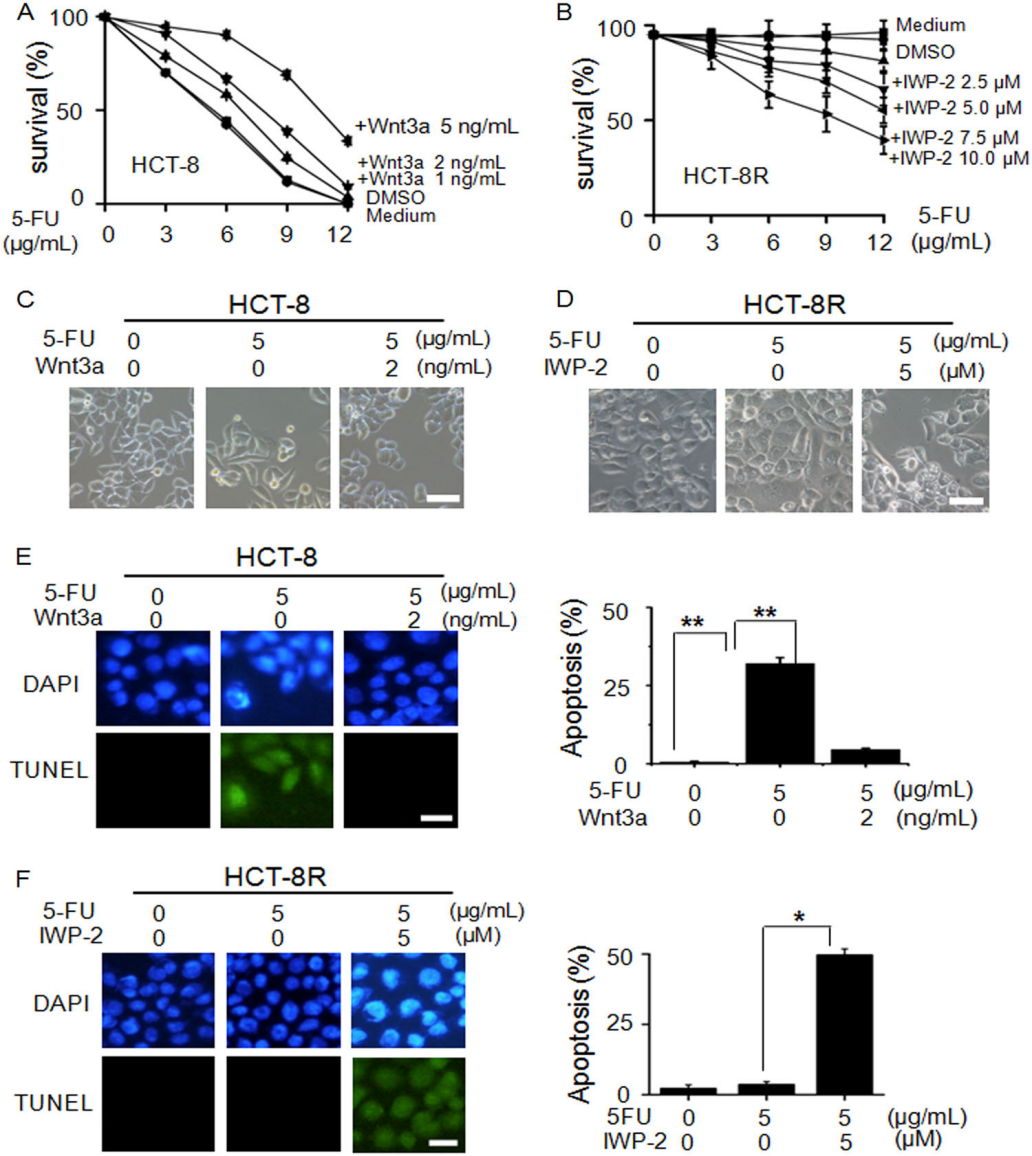
Regulation of the cell survival rate by Wnt activity in HCT-8R cells. **a** Wnt3a treatment improved the HCT-8 cell survival rate in 5-FU-containing medium. **b** Inhibition of the Wnt pathway decreased the cell viability of HCT-8R cells. **c** The cell morphology of HCT-8 cells treated with Wnt3a. Scale bar, 100 µm. **d** The cell morphology of HCT-8R cells treated with Wnt inhibitor. Scale bar, 100 µm. **e** TUNEL assay proved the enhanced resistance of Wnt3a-treated HCT-8 cells in 5 µg/mL 5-FU-contained medium. Scale bar, 20 µm. **P < 0.01. **f** TUNEL assay proved the apoptosis of HCT-8R cells induced by Wnt inhibitor. Scale bar, 20 μm. *P < 0.05

As shown in Fig. 5a, Wnt3a stimulation enhanced drug resistance in a dose-dependent manner. In contrast, HCT-8R cells became less drug resistant as the dosage of IWP-2 increased (Fig. 5b). The typical morphology of HCT-8 cells under treatment with Wnt3a and HCT-8R cells under the treatment with IWP-2 are shown in Fig. 5c, d, respectively. The accumulated data demonstrated that the alteration of Wnt pathway activity leads to a change in drug resistance in HCT-8 and HCT-8R cells.

To confirm the previous data, we performed TUNEL assays to examine the apoptotic cells. As shown in Fig. 5e, TUNEL assays indicated that the HCT-8 cells stimulated by Wnt3a acquired resistance to drug-induced apoptosis. In Fig. 5f, TUNEL assay of the control group showed that the Wnt inhibitor treatment restored cell sensitivity to 5-FU treatment. Consistent with the above data, the results of immunofluorescence staining of phospho-CHK1, phospho-p53, and β-catenin are shown in Figure S5A (HCT-8 cells stimulated by Wnt3a) and Figure S5B (HCT-8R cells under the treatment of IWP-2). The induction of P53 and CHK1 was further confirmed by western blotting (Figure S5C). The accumulative data verifies that the Wnt pathway suppresses the CHK1 pathway to inhibit 5-FU-induced apoptosis in HCT-8R cells.

### Xenograft experiments in a nude mouse model

To validate our findings, we conducted a xenograft experiment in a mouse model. HCT-8R or HCT-8 cells were injected into the mice. Ten days later, drugs were given intraperitoneally daily for 5 consecutive days. Then, cancer growth inhibition was monitored for up to 30 days. The results showed tumor volume growth in the control group (injected with PBS or 5-FU only), and Wnt3a significantly accelerated HCT-8 cancer cell growth under 5-FU treatment. This suggests that Wnt pathway stimulators can enhance drug resistance; however, the Wnt inhibitor IWP-2 significantly suppressed HCT-8R cancer growth. While the tumor volume increased in the control group (injected PBS or 5-FU only), it decreased under the combined treatment with the Wnt pathway inhibitor IWP-2 and 5-FU, suggesting that the combination treatment can enhance the therapeutic efficacy of chemotherapy drugs in a xenograft cancer model (Figure S6A and S6B). These above results were supported by biochemical analysis of tumor slides, as shown in Figure S6C-F.

### Histone deacetylation mediates the regulation of Wnt-CHK1

**Fig. 6:**
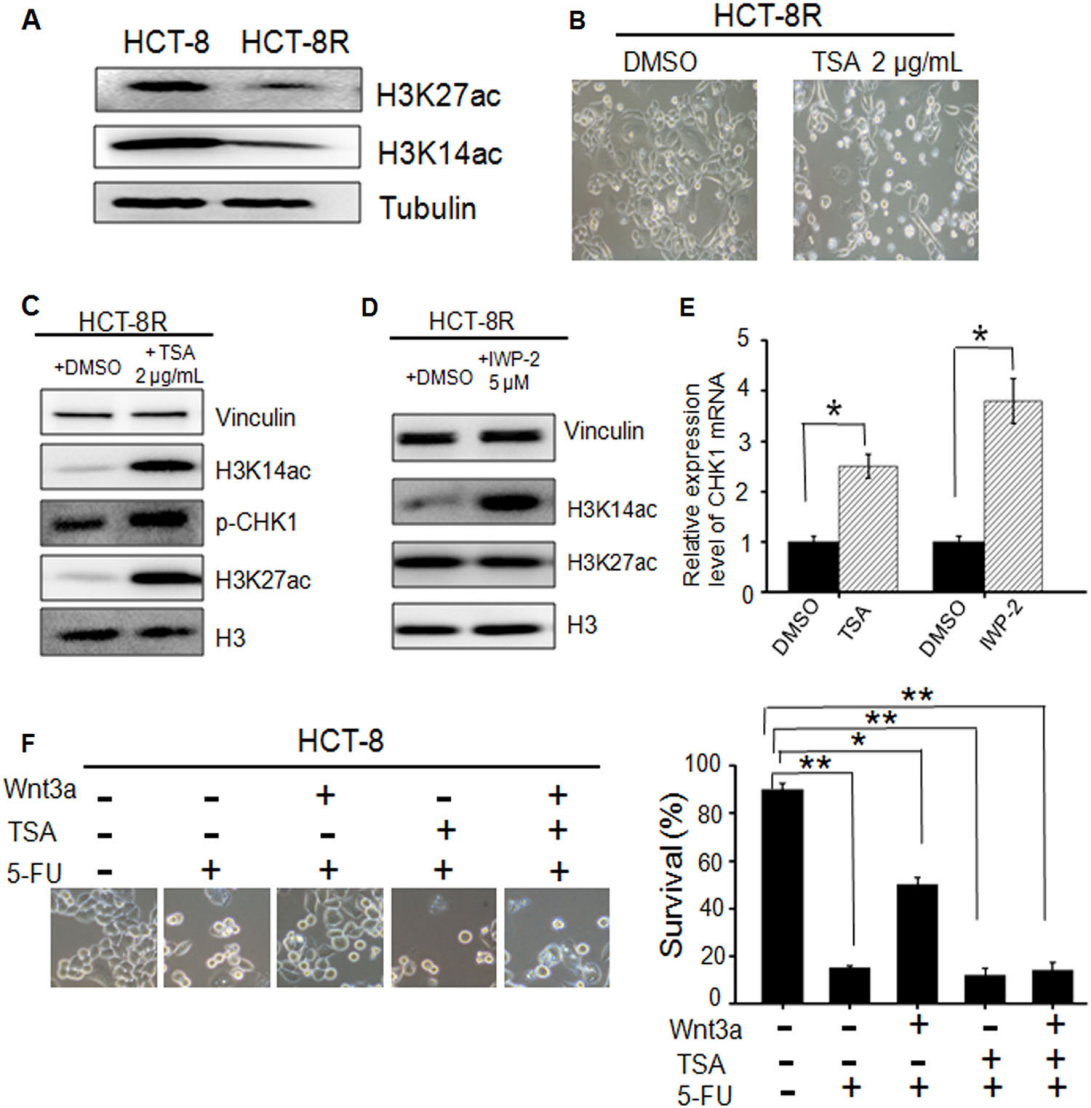
Histone modification is involved in Wnt-mediated regulation of CHK1. **a** Histone acetylation status was different in HCT-8 and HCT-8R cells. Shown are the western blotting assays of H3K27ac and H3K14ac expression level. **b** TSA, a well-established histone deacetylase (HDAC) inhibitor, sensitized HCT-8R cells to 5-FU treatment. **c** Western blotting assay of HCT-8R cells treated with TSA. **d** Western blotting assay of HCT-8R cells treated with the Wnt signal inhibitor IWP-2. **e** CHK1 mRNA level in HCT-8R cells treated with IWP-2 or TSA. *p < 0.05. **f** Cell survival of HCT-8 cells treated with Wnt3a or TSA. **p < 0.01

**Fig. 7:**
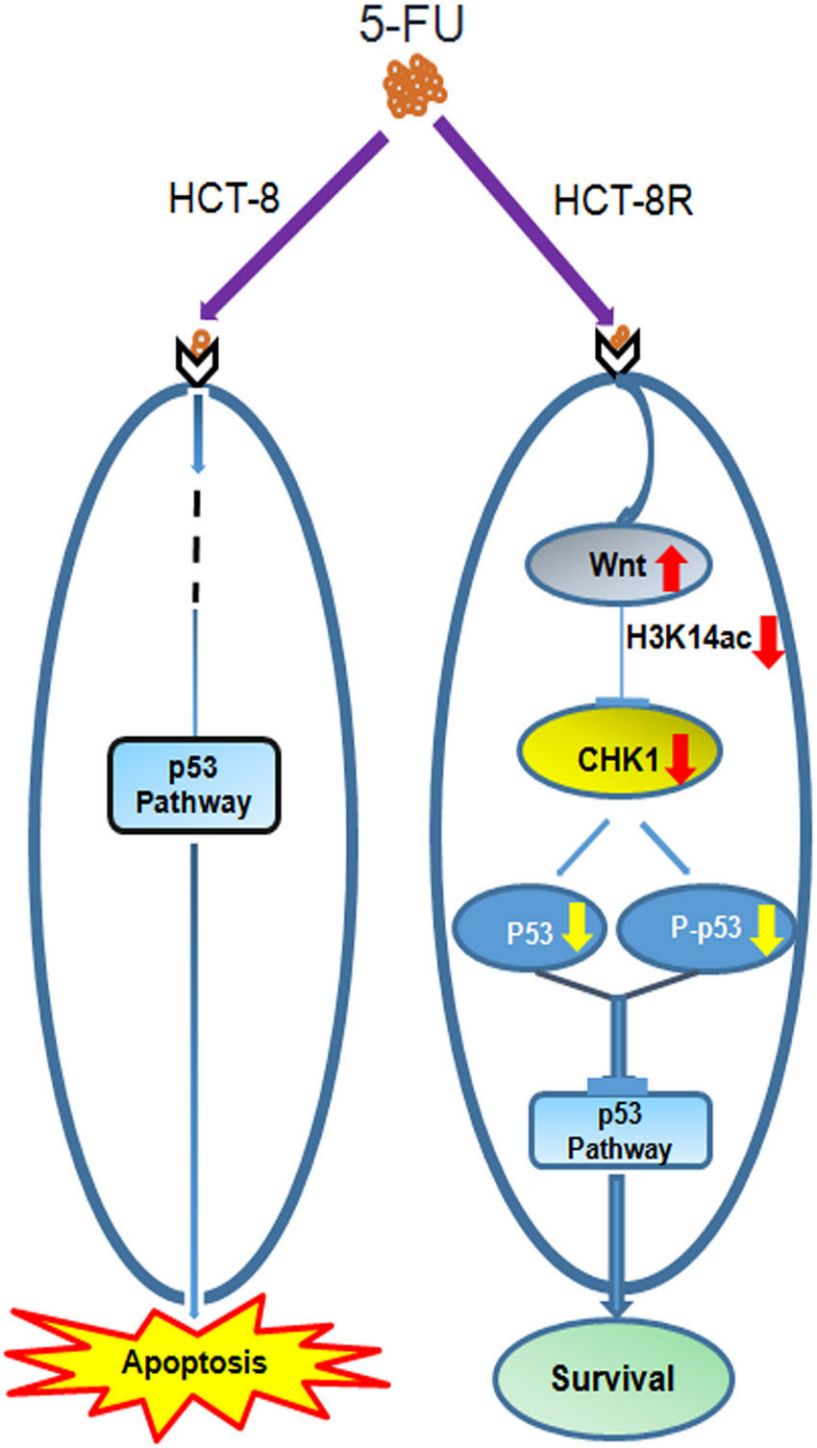
A schematic representation of HCT-8R cell resistance to 5-FU. A schematic representation showing that the p53 pathway is normal and responsive to proapoptotic reagents such as 5-FU in HCT-8 cells, but in 5-FU-resistant colorectal cancer cells (HCT-8R) with a wild-type p53 pathway, the Wnt pathway suppresses CHK1-induced cell cycle arrest and apoptosis through inhibition of the CHK1 pathway, which phosphorylates and stabilizes p53. H3K14ac modification plays a critical role in the Wnt-mediated regulation of drug resistance

To reveal the crosstalk mechanism between Wnt signaling and the CHK1 pathway, we compared the histone modification differences between HCT-8 and HCT-8R cells. As shown in Fig. 6a, 5-FU-resistant cells exhibited histone deacetylation on H3K14 and H3K27. TSA treatment^27^ sensitized HCT-8R cells to 5-FU treatment (Fig. 6b), suggesting that histone acetylation/deacetylation^28^ plays a critical role in the regulation of drug resistance. To confirm the above data, the expression levels of H3K14ac and H3K27ac in HCT-8R cells were examined after treatment with TSA (Fig. 6c) or IWP-2 (Fig. 6d). The results proved that H3K14ac but not H3K27ac was the key acetylation/deacetylation site involved in the Wnt-mediated regulation of drug resistance. Other representative acetylation sites on histone H3 or H4 were also checked, such as H3K9, but no obvious changes were observed (data not shown). The above data are further proved by the mRNA-level analysis (Fig. 6e). Next, to study whether Wnt-mediated regulation of drug resistance was acetylation/deacetylation-dependent, we performed a drug sensitivity assay. As shown in Fig. 6f, the cell survival assay suggested that the deacetylation of histone H3 was indispensable to Wnt-mediated regulation of drug resistance in HCT-8R cells. All these above data revealed a novel drug-resistant mechanism mediated by Wnt signal, as summarized in Fig. 7.

## Discussion

Wnt/β-catenin signaling is reportedly involved in a multitude of developmental processes, regulating cell proliferation, differentiation, migration, genetic stability, and apoptosis. Although therapeutic agents specifically targeting the Wnt pathway have recently entered clinical trials, the detailed mechanism of the Wnt pathway and how it generates chemotherapeutic drug resistance is still unclear^29^. Activation of canonical Wnt signaling is frequently studied in cancer; however, the links between the Wnt pathway and the p53-induced apoptotic pathway remain unclear. The mechanism behind the interaction of the Wnt pathway with CHK1 and the apoptotic response of cells to the chemical therapeutic drugs is also not well understood.

In this study, we generated and identified a panel of 5-FU-resistant cell lines from HCT-8 cells, providing a tool to investigate the molecular pathways and detailed mechanisms that may be associated with drug resistance in CRC. Although HCT-8 is a cancer cell line, it displays a wild-type TP53 phenotype, which means the p53 pathway is normal and responsive to proapoptotic reagents such as 5-FU, as shown in our data. Our work proved that in 5-FU-resistant CRC cells with a wild-type p53 pathway, the Wnt pathway suppresses CHK1-induced cell cycle arrest and apoptosis through the inhibition of the CHK1 pathway, which subsequently phosphorylates and stabilizes p53. These findings reveal crosstalk between the Wnt pathway and checkpoint control via the regulation of CHK1 (Fig. 6). Our paper suggests that in p53-normal cancer cells, crosstalk between Wnt signaling and the CHK1 pathway is a potential target for clinical therapy (Fig. 7).

In p53-deficient cancer cells, the mechanism appears to be completely different^30^. G1 arrest is defective in many cancers. Because more than 60% of cancers are defective in the ep53 pathway, the CHK1-dependent G2 checkpoint is a major target for chemotherapy^31^. In this regard, CHK1 inhibition increases the sensitivity of p53-deficient cancer cells to DNA damage agents^32^. Interestingly, CHK1 expression is also higher in p53-deficient cancer. It is suggested that relatively high levels of CHK1 may favor cell proliferation in p53-deficient cancer^33^.

P53 mutation or functional loss will accelerate the cancer progression generated by aberrant Wnt signaling. In a mutation-induced premalignancy mouse model, APC coupled with the TP53 deletion induces mucinous cystic neoplasms and invasive pancreatic carcinoma^34,35^. Using a p53-deficient transgenic mouse model, a study on cholangiocarcinoma (CC) determined that the Wnt pathway is highly activated in CCs, suggesting that targeting Wnt signaling pathways has potential as a therapeutic strategy for cancers that can be pharmacologically inhibited^36,37^. On the other hand, p53 has been reported to regulate the Wnt pathway. Recent evidence indicates that the microRNA-34 family, a group of transcriptional targets of p53, directly suppresses a set of canonical Wnt genes and Snail, which leads to p53-mediated suppression of Wnt signaling^38,39^.

## Electronic supplementary material

Figure S1. BER pathway analysis in resistant cells

Figure S2. Diagram of pathway analysis

Figure S3. P53 expression in drug resistant cells and its regulation by CHK1

Figure S4. The immunofluorescence staining result

Figure S5. Induction of P53 and CHK1 expression in HCT-8 or HCT-8R cells

Figure S6. The results of animal model

Supplementary Table 1

Supplementary Materials
