# Supplementary figures and images for: Wnt pathway is involved in 5-FU drug resistance of colorectal cancer cells
Source: Exp Mol Med. 2018 Aug 14;50(8):101. doi: 10.1038/s12276-018-0128-8 (PMC6093888; doi:10.1038/s12276-018-0128-8)

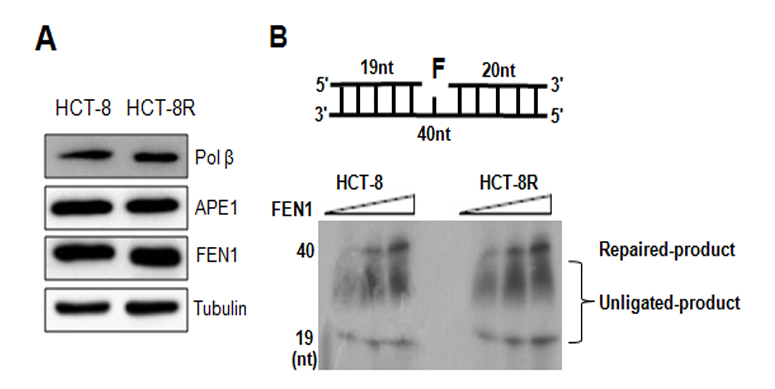

Supplement: Supplementary file 1 — Figure S1. BER pathway analysis in resistant cells [file 12276_2018_128_MOESM1_ESM.jpg]

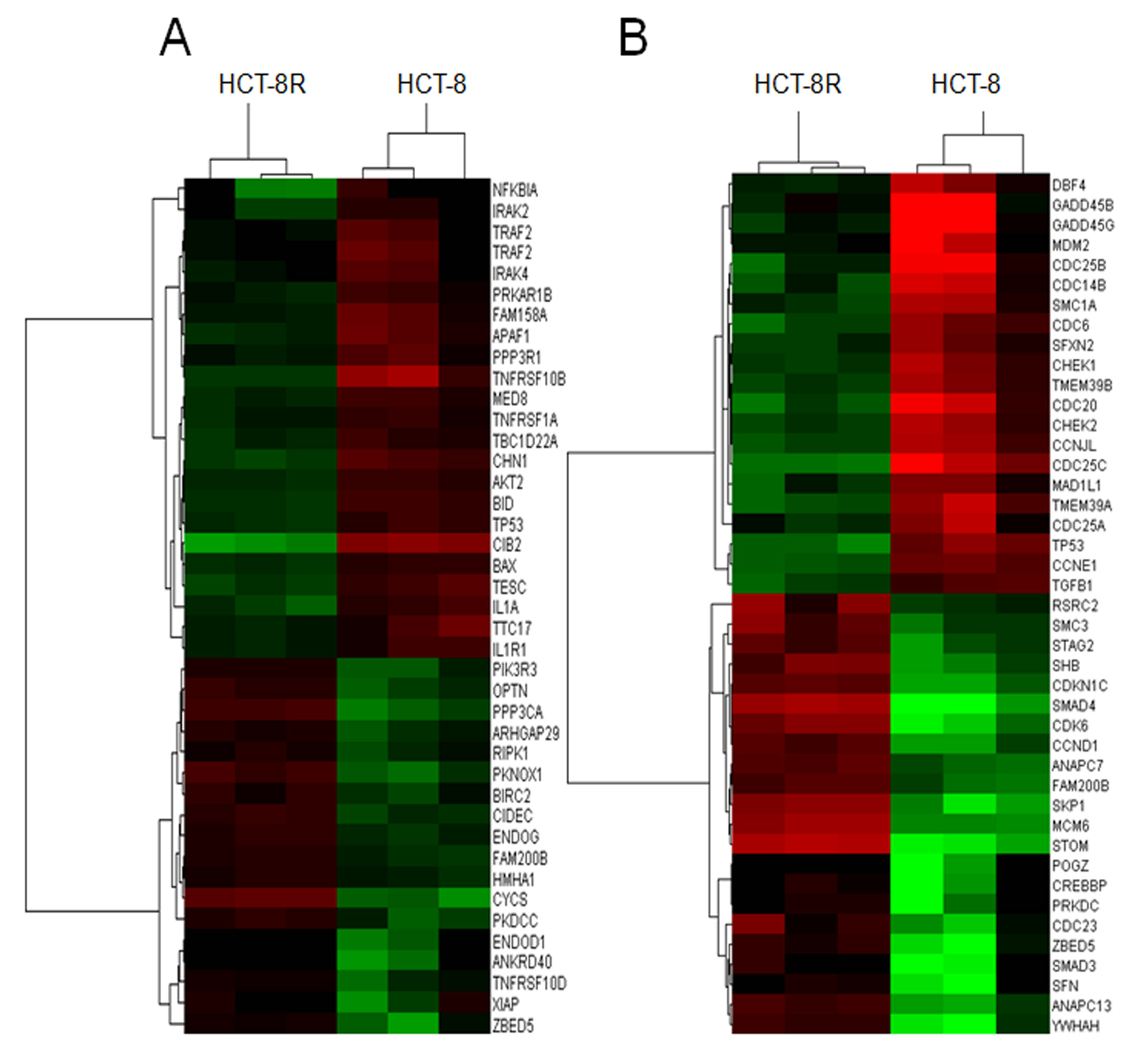

Supplement: Supplementary file 2 — Figure S2. Diagram of pathway analysis [file 12276_2018_128_MOESM2_ESM.jpg]

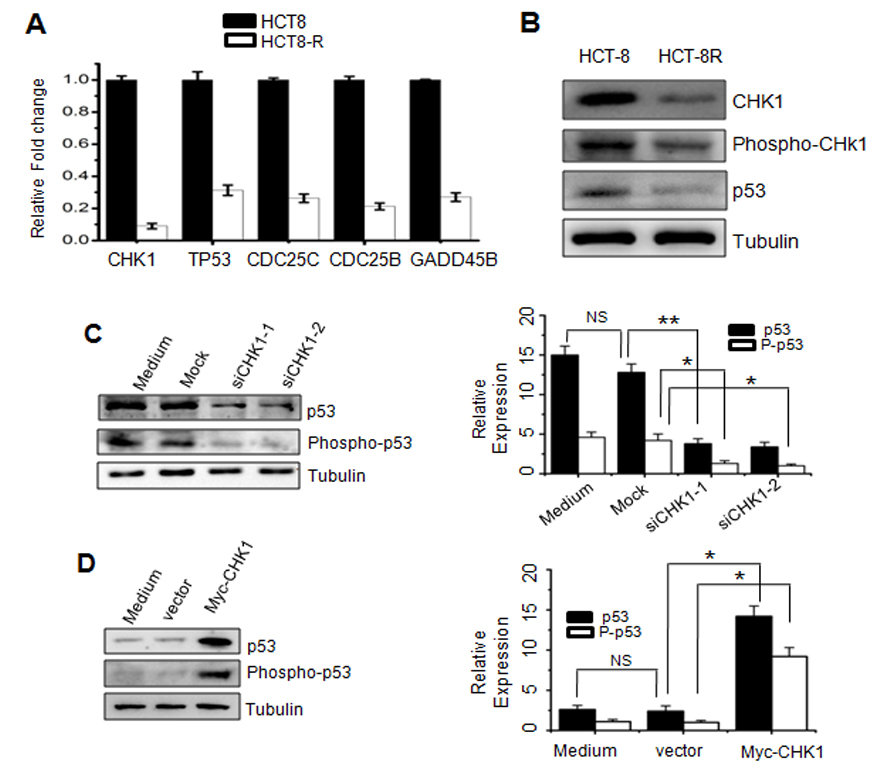

Supplement: Supplementary file 3 — Figure S3. P53 expression in drug resistant cells and its regulation by CHK1 [file 12276_2018_128_MOESM3_ESM.jpg]

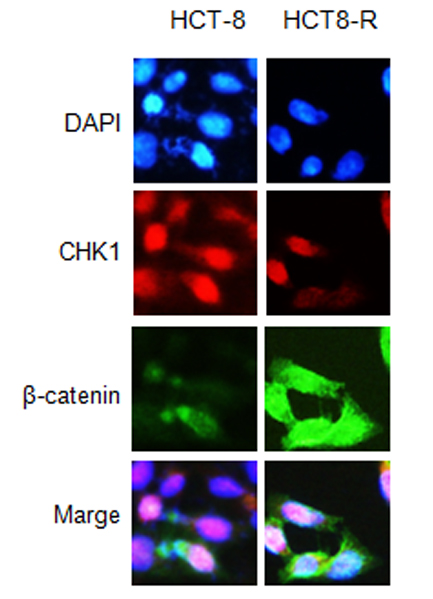

Supplement: Supplementary file 4 — Figure S4. The immunofluorescence staining result [file 12276_2018_128_MOESM4_ESM.jpg]

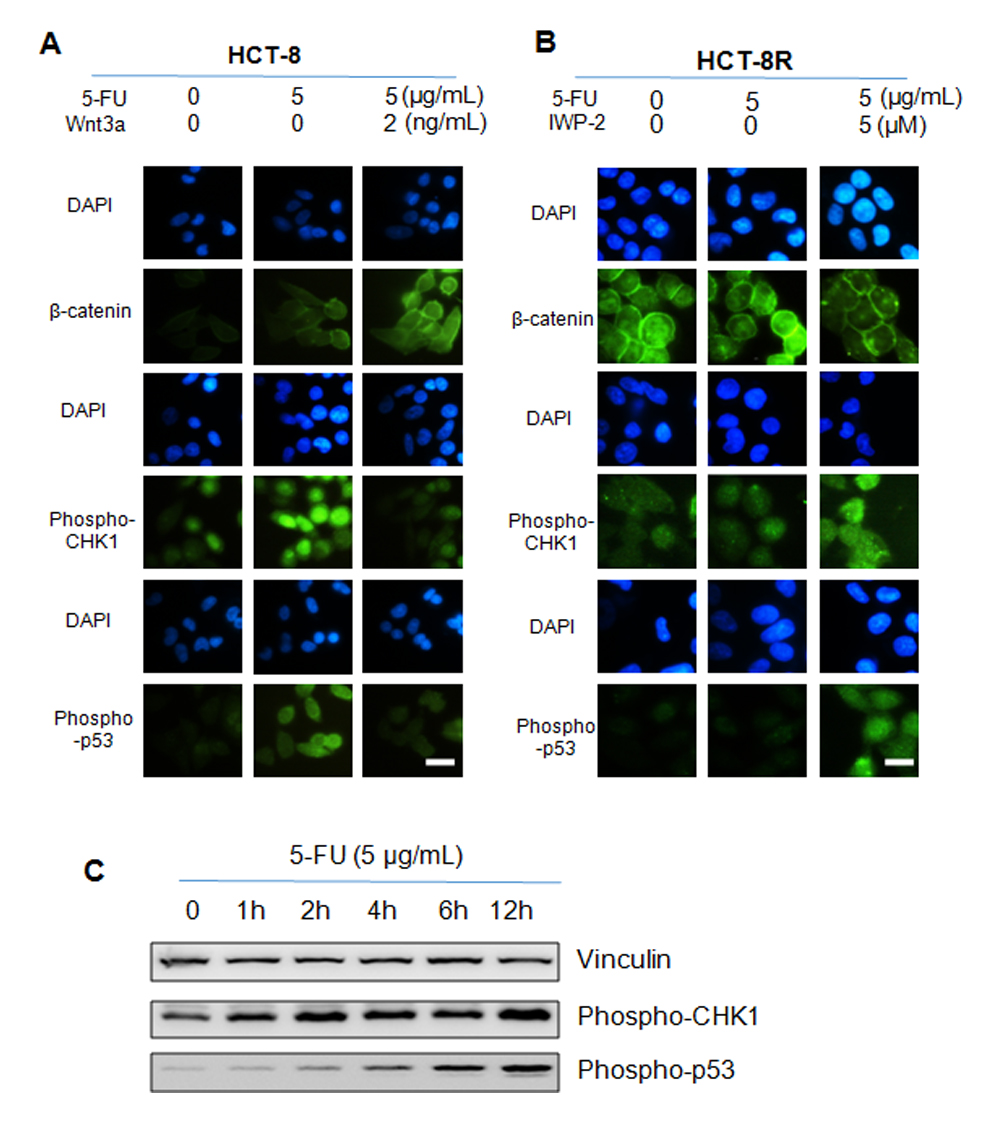

Supplement: Supplementary file 5 — Figure S5. Induction of P53 and CHK1 expression in HCT-8 or HCT-8R cells [file 12276_2018_128_MOESM5_ESM.jpg]

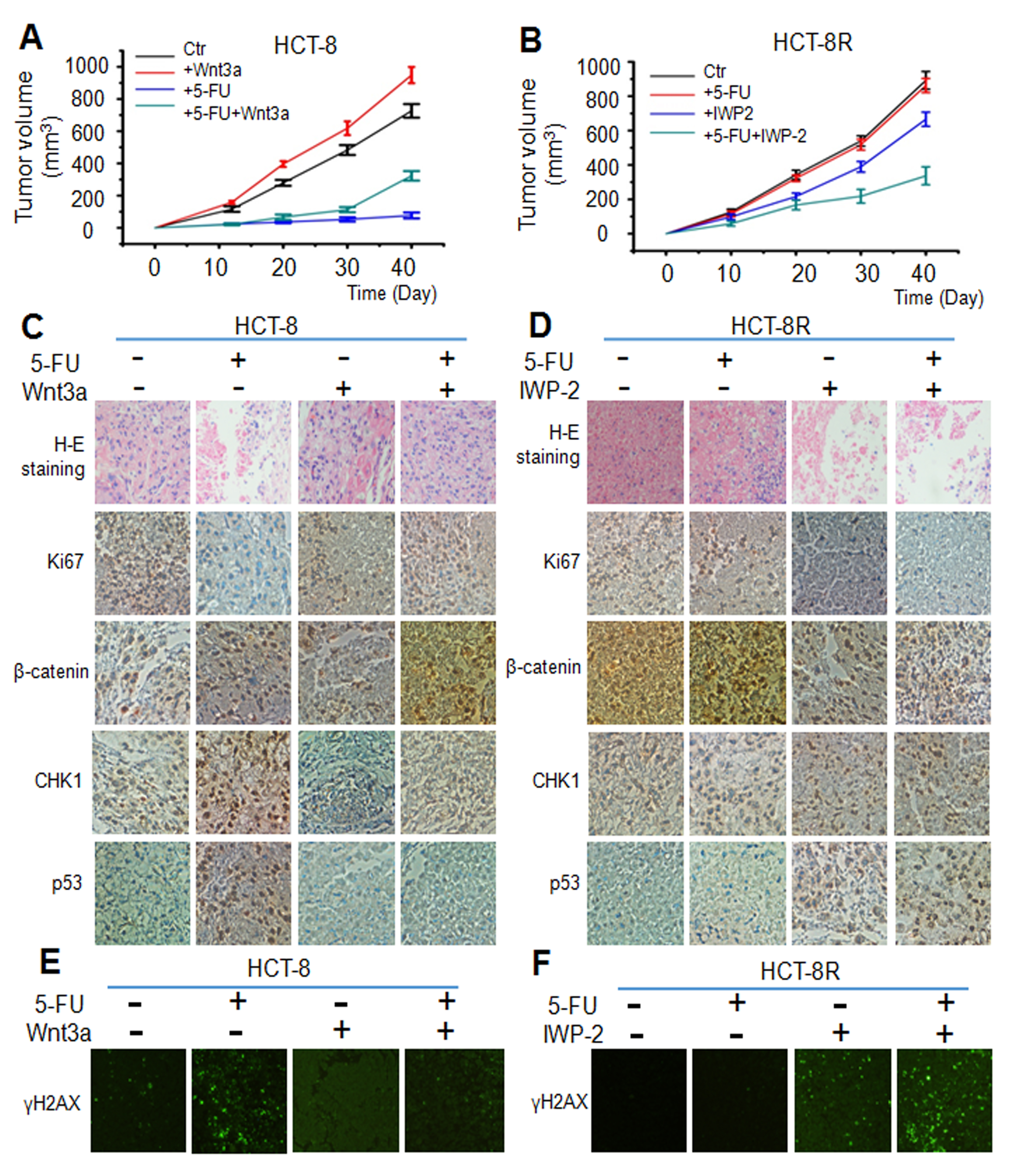

Supplement: Supplementary file 6 — Figure S6. The results of animal model [file 12276_2018_128_MOESM6_ESM.jpg]

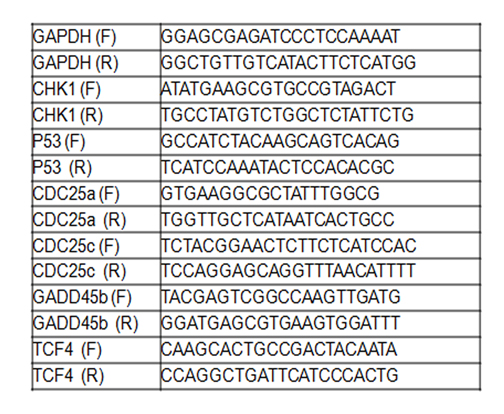

Supplement: Supplementary file 7 — Supplementary Table 1 [file 12276_2018_128_MOESM7_ESM.jpg]
