# Supplementary material for: Wnt pathway is involved in 5-FU drug resistance of colorectal cancer cells
Source: Exp Mol Med. 2018 Aug 14;50(8):101. doi: 10.1038/s12276-018-0128-8 (PMC6093888; doi:10.1038/s12276-018-0128-8)
Supplement: Supplementary file 8 — Supplementary Materials [file 12276_2018_128_MOESM8_ESM.docx]

**Figure S1. BER pathway analysis in resistant cells.**

(A) The expression of APE1, FEN1 and Polβ in the HCT-8 and HCT-8R cells were examined by western blotting assay. (B) The DNA repair ability assay of HCT-8 and HCT-8R cells was compared using LP-BER assay.

**Figure S2. Diagram of pathway analysis.**

1. Cell cycle. (B) Apoptosis pathway.

**Figure S3. P53 expression in drug resistant cells and its regulation by CHK1.**

(A) Confirmation of expression pattern of HCT-8R cells by quantitative RT-PCR of the selected gene from the gene list to testify the microarray data. (B) The expression of p53 and CHK1 were re-checked by western blotting assay in the wild type HCT-8 cells and 5-FU resistant cells. (C) The regulation of p53 signal by CHK1 was also confirmed by CHK1 knockdown in the HCT-8 cells. The transfected CHK1 siRNA efficiently decreased the expression of p53 and their phosphorylated form. (D) The regulation of p53 signal by CHK1 was confirmed by CHK1 over-expression in HCT-8R cells. Over-expressed CHK1 improved p53 expression drastically in HCT-8R cells under 5-FU treatment.

**Figure S4. The immunofluorescence staining result.**

Shown are the expressions of β-Catenin and CHK1 protein in HCT-8 and HCT-8R cells.

**Figure S5. Induction of P53 and CHK1 expression in HCT-8 or HCT-8R cells.**

Shown are the expression of phosphorylated p53, CHK1 and β-catenin in (A) HCT-8 and (B) HCT-8R cells under different treatments. (C) Western blotting assay of phosphorylated p53 and CHK1 expression in HCT-8 cells after 5-FU treatment.

**Figure S6. The results of animal model.**

(A) HCT-8 cells (2x10^6^ cells) were subcutaneously implanted in the right flank of nude mice. Treatment began when the average tumor size reached 1 mm^3^. Mice were randomly allocated to four groups, namely control, Wnt3a (100 µg/kg), 5-FU (100 mg/kg), and Wnt3a (100 µg/kg) combined with 5-FU (100 mg/kg). After consecutive treatment, the tumor size was measured twice a day by Vernier caliper after the initiation of drug treatment. (B) HCT-8R (2x10^6^ cells) was subcutaneously implanted in the right flank of nude mice. Treatment began when the average tumor size reached 1 mm^3^. Mice were randomly allocated to four groups, namely control, IWP-2 (200 mg/kg), 5-FU (100 mg/kg), and IWP-2 (200 mg/kg) combined with 5-FU (100 mg/kg). After consecutive treatment, the tumor size was measured twice a day by Vernier caliper after the initiation of drug treatment. The data represent the means ± SD from three independent experiments. *P <0.05. (C) and (D) are H-E staining and immunochemistry analysis. (E) and (F) are immunofluorescenent staining of γH2AX.

**Supplemental Table 1. The primer sequence.**

Shown are the primer sequences of Q-PCR used in this paper.
